# Investigation of the Response of Binary Multileaf Collimator Compensation to Target Setup Errors in the Radixact Synchrony System: A Phantom Study

**DOI:** 10.7759/cureus.85305

**Published:** 2025-06-03

**Authors:** Masatoshi Miura, Koji Sasaki, Yasuo Shiota, Kazuyasu Inoue

**Affiliations:** 1 Department of Medical Physics, Iwata City Hospital, Iwata, JPN; 2 Graduate School of Radiological Technology, Gunma Prefectural College of Health Sciences, Maebashi, JPN; 3 Department of Radiation Technology, Iwata City Hospital, Iwata, JPN

**Keywords:** dosimetric accuracy, motion management, radixact, real-time adaptive radiotherapy, synchrony, tomotherapy

## Abstract

The Radixact Synchrony system incorporates real-time target tracking into helical tomotherapy, dynamically correcting motion via jaw tracking and binary multileaf collimator (MLC) offset adjustments. Jaw tracking offers adjustments for longitudinal motion (IEC-Y), whereas MLC-based compensation for transverse motion (IEC-X and IEC-Z) is constrained by the 6.25-mm discrete leaf width, potentially affecting dosimetric accuracy. Furthermore, to formulate effective therapeutic approaches in motion-adaptive radiation therapy, understanding the MLC compensation threshold is crucial. This study aimed to assess the robustness of setup error correction and estimate the binary MLC compensation threshold in the Radixact Synchrony system through a phantom-based dosimetric analysis.

Experiments were conducted using the Accuray Precision treatment planning system (version 3.3.1, Accuray, Sunnyvale, CA, USA) and the Radixact X9 (version 3.0.3, Accuray, Sunnyvale, CA, USA). A stereotactic body radiation therapy plan for lung cancer was delivered to a moving phantom using Radixact Synchrony. The phantom motion was simulated using the Delta4 Phantom+ and the Hexamotion stage. To evaluate the impact of setup errors on dosimetric accuracy, measurements were performed under longitudinal and transverse setup error conditions. Dosimetric accuracy was evaluated on the basis of the gamma pass ratio (GPR) and dose profile analysis, and the binary MLC compensation threshold was estimated.

Along the longitudinal direction, jaw tracking effectively preserved dosimetric accuracy despite setup errors. Along the transverse directions, the GPR decreased as setup errors increased but improved when MLC compensation became active. Dose profile analysis revealed that MLC compensation was initiated at 4.3 mm.

This study evaluated the impact of MLC compensation on dosimetric accuracy and estimated its threshold to be approximately 4.3 mm in the Radixact Synchrony system. Jaw tracking effectively corrects for longitudinal motion, whereas MLC-based compensation in the transverse direction is constrained by its discrete resolution and threshold activation. These findings offer valuable insights for refining treatment margins and enhancing motion-adaptive radiation therapy strategies. To enhance the accuracy and efficacy of Synchrony-based motion correction, studies focusing on clinical validation and parameter optimization are warranted.

## Introduction

Radixact (Accuray, Sunnyvale, CA, USA), a helical tomotherapy system, is designed to deliver high-precision intensity-modulated radiation therapy (IMRT). By enabling real-time target tracking during beam delivery and dynamically correcting motion using both the jaws and the binary multileaf collimator (MLC), the Radixact Synchrony system enhances treatment accuracy [1]. Longitudinal motion (IEC-Y, superior/inferior) is corrected through jaw tracking, whereas transverse motion (IEC-X, left/right; IEC-Z, anterior/posterior) is corrected by adjusting the planned MLC opening positions according to target displacement at each gantry angle. However, owing to the discrete nature of the MLC, with a 6.25-mm leaf width at the isocenter (IC), its correction accuracy is lower than that of the jaws, potentially affecting dosimetric accuracy.

The tracking accuracy and dosimetric performance of Radixact Synchrony have been extensively evaluated in phantom-based studies, which have demonstrated that Synchrony effectively mitigates motion-induced dose blurring and beam interplay effects, thereby maintaining dosimetric accuracy under respiratory motion [1-3]. Tracking accuracy is influenced by several factors, including correlation variations between target and surrogate motion, image acquisition frequency, and the respiratory tracking control threshold [4,5]. The system's correction accuracy relies on both the jaw and MLC adjustments, with previous research indicating that MLC-based corrections require a motion threshold exceeding 3.125 mm before activation [6]. Furthermore, the impact of variable jaw width on beam characteristics was evaluated, demonstrating its role in correcting for output variations due to jaw offset and enhancing dosimetric accuracy [7]. Chen et al. [2] reported that small setup errors can be corrected by Synchrony tracking, suggesting that setup margins may be reduced through effective Synchrony-based correction. However, their assessment focused primarily on corrections in the IEC-Y direction via jaw tracking and evaluated MLC-based compensation for relatively large displacements (e.g., ≥5 mm). As such, the system's behavior near the MLC compensation threshold in the transverse directions (IEC-X and Z) remains underexplored. While Ferris et al. [3] reported that motion interplay and dose blurring were not observed in any of the cases studied when Synchrony tracking was enabled, they did not address conditions involving small, persistent displacements such as setup errors that may not trigger MLC-based compensation.

Clinically, Synchrony has been explored for tumor-tracking radiation therapy across various anatomical sites, including the lung, liver, and prostate [8-11]. Studies have underscored the significance of patient selection, tracking parameter optimization, and the feasibility of reducing planning target volume (PTV) margins owing to enhanced motion correction. While intrafractional motion has been extensively studied, the role of initial setup errors, particularly how they affect the activation and efficacy of MLC corrections, remains underexplored. Since setup errors are often corrected manually prior to treatment but may persist within clinically acceptable margins, their interaction with automated tracking systems such as Synchrony has important implications for treatment accuracy.

This study aimed to clarify the MLC compensation threshold in Radixact Synchrony tracking by analyzing the gamma pass ratio (GPR) and dose distribution, thereby providing new insights into Radixact Synchrony's principles, and helping develop clinical treatment guidelines by examining the impact of setup errors and correction mechanisms on treatment accuracy.

## Technical report

Treatment planning and delivery

**Figure 1:**
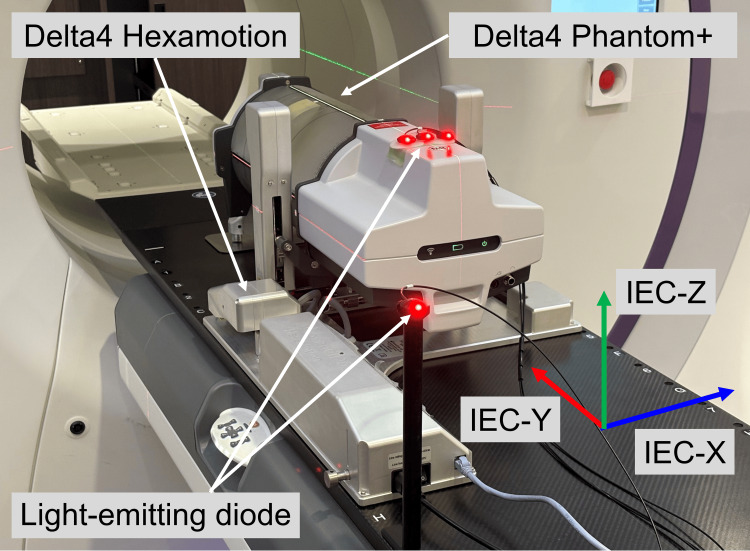
Experimental setup for dose distribution measurement using the Delta4 Phantom+ device placed on the Hexamotion stage to simulate respiratory motion The Delta4 Hexamotion system is employed for simulating linear target motion along the IEC-Y direction using a basic respiratory model (fourth power of the sinusoidal wave). The respiratory cycle is set to four seconds, and the amplitude corresponding to each cycle is 10 mm in the negative IEC-Y direction from the planned target position (isocenter in this study).

Treatment plans were developed using the Accuray Precision treatment planning system (version 3.3.1, Accuray, Sunnyvale, CA, USA), referred to as TPS, for stereotactic body radiation therapy for lung cancer. The treatment plans were executed using a tomotherapy system (Radixact X9, version 3.0.3, Accuray, Sunnyvale, CA, USA). Dosimetric verification was performed using the Delta4 Phantom+ and Hexamotion stage (both from ScandiDos, Uppsala, Sweden). The Delta4 Phantom+ system, comprising two orthogonal diode arrays, provided two-dimensional (2D) dose data per measurement, whereas the Hexamotion stage simulated translational motion along predefined trajectories (Figure 1).

Phantom imaging and treatment planning

Computed tomography images of the phantom were acquired using the Optima CT 580W (GE Healthcare, Waukesha, WI, USA) with the following parameters: tube voltage, 120 kV; field of view, 650 mm; and slice thickness, 1.25 mm. These images were imported into the TPS, where the phantom was aligned at the IC for treatment planning. A 3-cm-diameter spherical PTV was placed at the center of the Delta4 Phantom+. The diode array spacing within the Phantom+ measured 5 and 10 mm in the central and peripheral regions, respectively. To optimize measurement accuracy, the high-dose region was aligned with the high-resolution section of the diode array. To correct for dose attenuation properties, the physical density of the phantom was overridden to 1.160 g/cm³.

Radiation delivery parameters

**Figure 2:**
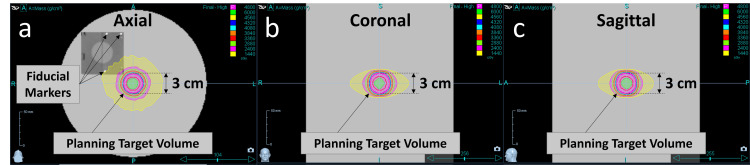
Dose distribution calculated on the Delta4 Phantom+ using the treatment planning system The prescribed dose is 12 Gy per fraction at 95% of the planning target volume. The following are the main calculation conditions: field width, 2.5 cm; pitch, 0.1; gantry rotation period, 18.7 seconds; and total treatment time, 363.4 seconds. The dose distribution is shown in the axial (a), coronal (b), and sagittal (c) planes.

Treatment planning was optimized using IMRT (Figure 2). The prescribed dose was 12 Gy/fraction, with 95% of the PTV covered by this dose. A 6-MV flattening filter-free photon beam was delivered at a dose rate of 1000 monitor unit (MU)/minute, with a 2.5-cm jaw setting and a pitch of 0.1. The gantry rotation period was 18.7 seconds, and the total treatment time was 363.4 seconds.

Setup error simulation

The Radixact Synchrony system was employed for tracking and correcting target motion, modeling the target offset relative to the planned position (IC). Setup errors were introduced by inputting specified translational displacements into the Hexamotion system, which then mechanically shifted the phantom along the IEC-X, IEC-Y, and IEC-Z directions with high precision. This method ensured reproducibility and eliminated manual manipulation. The phantom was systematically shifted along the IEC-X, IEC-Y, and IEC-Z directions in 1.0-mm increments from 1.0 to 5.0 mm to evaluate setup error correction accuracy. Additionally, to estimate the compensation threshold of the MLC, offsets ranging from 4.1 to 4.9 mm were applied in 0.1-mm increments along the IEC-X direction.

Motion tracking using the Radixact Synchrony system

Real-time motion tracking using fiducials with respiratory mode on the Radixact Synchrony system was used. Six gold fiducial markers (diameter, 0.8 mm; length, 5 mm) embedded within the Ball-Cube II (Accuray, Sunnyvale, CA, USA) inside the Delta4 Phantom+ were tracked using 2D kV radiographs. To generate surrogate respiratory signals, three light-emitting diode (LED) markers were affixed to the Phantom+, with an additional LED marker placed on a stationary object on the couch as a reference for the tracking algorithm. The internal fiducial positions extracted from the periodic 2D kV radiographs were correlated with the continuously monitored external LED marker positions. Based on this correlation, the system constructs an internal-external correlation model, which is dynamically updated during treatment to continuously estimate the 3D target position in real time [1].

Experimental conditions and dose verification

For dose quality assurance, the following three experimental scenarios were designed: (1) M0S0E0: Static phantom, Synchrony disabled, no setup error (baseline condition); (2) M1S1E0: Moving phantom, Synchrony enabled, no setup error; and (3) M1S1E1: Moving phantom, Synchrony enabled, with a setup error.

M0S0E0 served as the baseline for evaluating the response of the Radixact Synchrony to target motion and setup errors and its effect on dosimetric accuracy. Discrepancies in dose distribution observed under other conditions would indicate deviations from the baseline distribution in M0S0E0.

During treatment setup, kVCT was performed to identify internal fiducials and align the phantom with the planning image. The residual setup error after alignment was negligible, as the internal fiducials were clearly visualized and precisely matched to their planned positions. The Hexamotion system simulated target motion exclusively along the IEC-Y direction using a basic respiratory model (fourth power of a sinusoidal wave), mimicking respiratory-induced tumor motion. Respiratory cycles were set to 4 seconds, with a 10-mm amplitude in the negative IEC-Y direction. 2D kV radiographs were acquired at fixed gantry angles of 55°, 120°, 170°, 230°, 300°, and 350° during each 18.7-second gantry rotation using chest imaging parameters (1.6 mAs and 120 kV). Given the gantry rotation speed, this corresponded to approximately one image every 3.1 seconds. The thresholds for the parameters controlling tracking delivery were set to 2.0 mm for potential difference and 4.0 mm for measured delta. These parameters were used to determine tracking accuracy in the Synchrony system, and both the potential difference and measured delta were no greater than 0.5 mm. The Delta4 software automatically separated the imaging dose from the treatment dose, ensuring that kV radiographs have minimal impact on the measured doses. Specifically, the software identifies imaging dose by its broader spatial profile across the detectors. Dose packages containing only imaging dose are discarded, while mixed packages are corrected by subtracting the broad component beneath the treatment peak. This approach effectively minimizes the diode array's over-response to kV imaging.

Dose measurement and analysis

Using the Delta4 Phantom+, dose distributions were measured in the coronal and sagittal planes. Each measurement was performed once for each setup error condition. Although repeated measurements were not performed, all experiments were conducted under tightly controlled conditions to ensure experimental consistency and reproducibility. Furthermore, after the measurements in this study, we analyzed the recorded raw tracking log data, extracted the predicted tracked target positions, and compared them with the known setup errors, confirming that the position deviations during irradiation were minimal. Dosimetric accuracy was evaluated on the basis of the GPR and dose profile analysis. M0S0E0 served as the baseline for comparison with M1S1E0 and M1S1E1. GPR analysis was performed using a 3%/1-mm criterion, evaluating dose points receiving at least 10% of the maximum dose. This stricter distance-to-agreement (DTA) threshold was selected in consideration of the high precision required in stereotactic body radiotherapy delivery and to better detect subtle discrepancies in the dose distribution. To evaluate dose distribution patterns, one-dimensional dose profiles were extracted along the IEC-X, IEC-Y, and IEC-Z directions. All dose analyses were performed using the Delta4 software.

Dosimetric accuracy with Radixact Synchrony tracking (M0S0E0 vs. M1S1E0)

**Figure 3:**
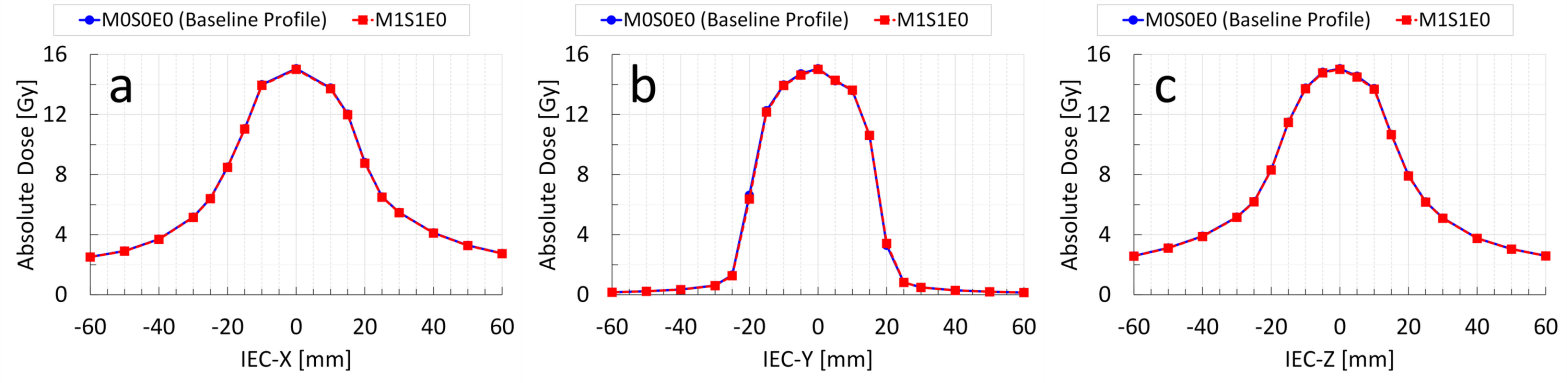
Comparison of measured dose profiles through the center of the Delta4 Phantom+ in the static baseline and moving phantom conditions using the Radixact Synchrony—M0S0E0 versus M1S1E0 The blue solid line represents the baseline dose profile (M0S0E0) measured along the central axis of the Delta4 Phantom+ for the static phantom with the Radixact Synchrony disabled and no setup error, whereas the red dashed line indicates the dose profile measured along the same axis for the moving phantom with the Radixact Synchrony enabled and no setup error (M1S1E0). Dose profiles along the IEC-X, IEC-Y, and IEC-Z directions are shown in (a), (b), and (c), respectively. These profiles demonstrate the consistency of the measured dose distributions when Synchrony is enabled without setup error.

To evaluate the performance of Synchrony tracking, the measured dose distributions for the moving phantom condition with Synchrony enabled (M1S1E0) were compared with the static baseline (M0S0E0). The GPR of M1S1E0 (setup error = 0 mm) relative to that of M0S0E0 was 100% under the 3%/1-mm criterion and evaluation of points receiving at least 10% of the maximum dose. Moreover, the dose profile comparison confirmed the consistency of the measured dose distributions when Synchrony was enabled without setup errors (Figure 3). In addition, comparison of the predicted tracked target positions from the tracking log data analysis with the known setup errors showed that the mean ± standard deviation of the positional deviation was 0.01 ± 0.05 mm for IEC-X, −0.03 ± 0.04 mm for IEC-Z, and 0.19 ± 0.13 mm for IEC-Y, indicating that the positional deviation during irradiation was very small.

Impact of setup errors (M0S0E0 vs. M1S1E1)

**Figure 4:**
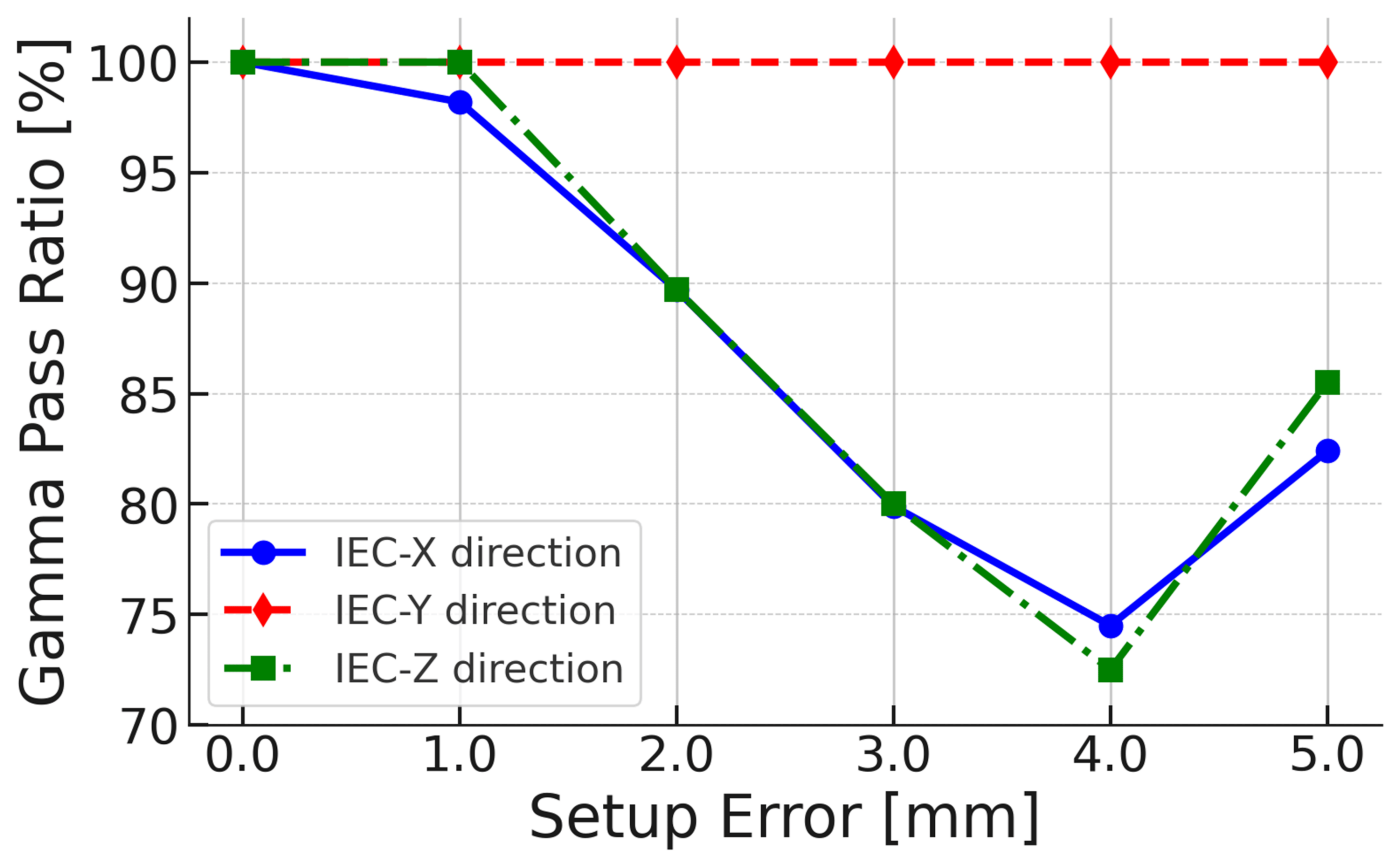
Change in Radixact Synchrony's response to target setup errors along the IEC-X, Y, and Z directions: gamma pass ratio (GPR)—M0S0E0 versus M1S1E1 Gamma analysis used the 3%/1-mm criterion with a 10% dose threshold. The blue solid line with circles, red dashed line with diamonds, and green dashed–dot line with squares represent the IEC-X, IEC-Y, and IEC-Z directions, respectively. In the IEC-Y direction, where setup errors are corrected by jaw tracking, the GPR remains at 100% under all conditions, confirming the accuracy of jaw-based correction. In contrast, in the IEC-X and IEC-Z directions, where binary MLCs provide compensation, the GPR decreases as the setup error increases, decreasing below 75% at 4.0 mm. However, the GPR improves when the setup error reaches 5.0 mm. These results indicate that MLC compensation is activated at a setup error of at least 5.0 mm.

The impact of setup errors on Synchrony tracking accuracy and dose distribution was evaluated by introducing intentional misalignments (M1S1E1). The GPR under the 3%/1-mm criterion and evaluation of points receiving at least 10% of the maximum dose demonstrated variation depending on the setup error direction and magnitude (Figure 4).

**Figure 5:**
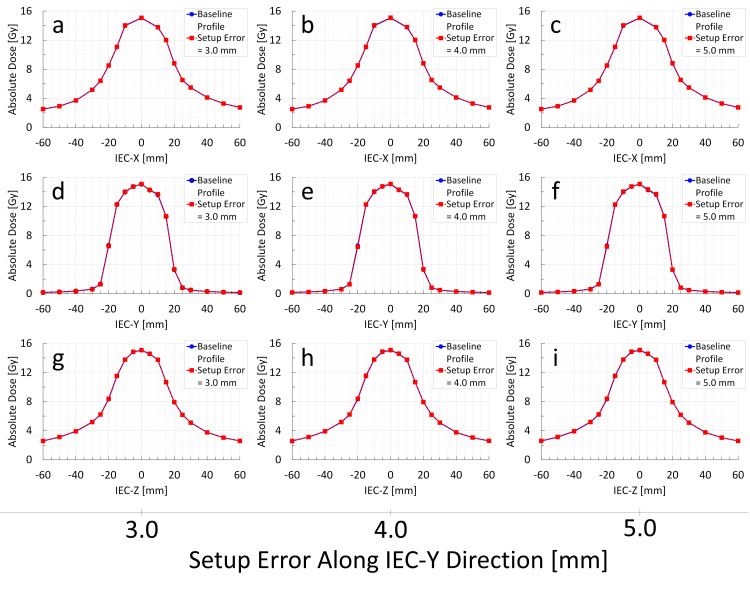
Change in Radixact Synchrony's response to target setup errors along the IEC-Y direction: analysis of measured dose profile variations through the center of the Delta4 Phantom+ (M0S0E0 vs. M1S1E1) The blue solid line indicates the baseline dose profile (M0S0E0), whereas the red dashed line represents the dose profile of Radixact Synchrony irradiation in response to a target setup error along the IEC-Y direction (M1S1E1). Little to no changes are observed in the IEC-X (a–c), IEC-Y (d–f), and IEC-Z (g–i) profiles, indicating minimal impact of target setup errors along the IEC-Y direction.

Along the IEC-Y direction, where correction was applied by the jaw system, the GPR remained 100% for all setup error conditions (Figure 4). Consistently, despite the presence of setup errors, the dose profile showed minimal deviation (Figure 5).

**Figure 6:**
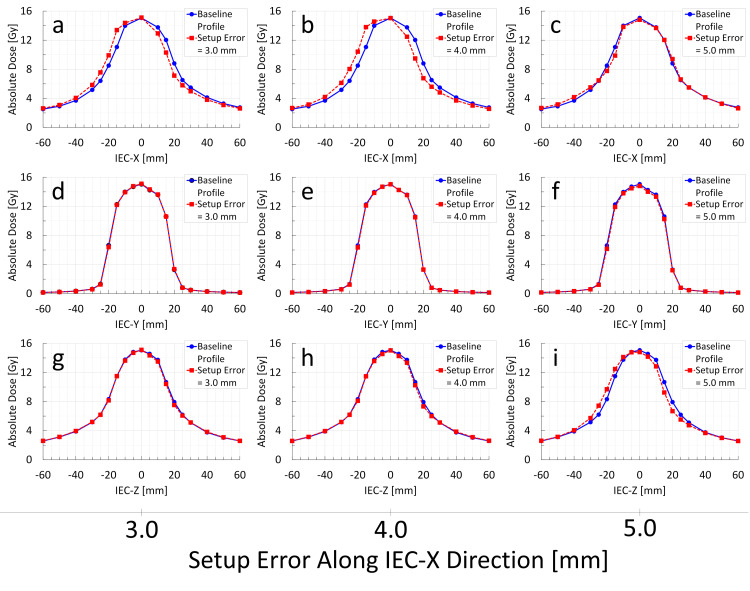
Change in Radixact Synchrony's response to target setup errors along the IEC-X direction: analysis of measured dose profile variations through the center of the Delta4 Phantom+ (M0S0E0 vs. M1S1E1) The blue solid line indicates the baseline dose profile (M0S0E0), whereas the red dashed line represents the dose profile of Radixact Synchrony irradiation in response to a target setup error along the IEC-X direction (M1S1E1). The observed changes in the dose profile indicate that the IEC-X profile is corrected when the setup error reaches 5.0 mm (a–c). Despite no setup errors observed in the IEC-Z direction, a profile shift is noted in IEC-Z when the target setup error in IEC-X reaches 5.0 mm (i). The IEC-Y profile shows minimal changes (d–f). These findings suggest that binary MLC compensation in one direction can influence dose profiles in the orthogonal direction, thereby altering the overall axial dose distribution.

In contrast, along the IEC-X direction, where compensation was performed using the MLC, the GPR was 98.2% for a 1.0-mm setup error and decreased to 89.7%, 79.9%, and 74.5% for 2.0-, 3.0-, and 4.0-mm setup errors, respectively, before slightly improving to 82.4% at 5.0 mm (Figure 4). Similarly, along the IEC-Z direction, the GPR was 100% at 1.0 mm but dropped to 89.7%, 80.0%, and 72.5% for 2.0-, 3.0-, and 4.0-mm setup errors, respectively, before improving to 85.5% at 5.0 mm (Figure 4). The observed GPR improvement at a setup error of at least 5.0 mm suggests MLC compensation activation. This finding is further supported by dose profile analysis results, revealing that dose profile deviations persisted for setup errors along the IEC-X direction of up to 4.0 mm, indicating a not fully engaged MLC compensation (Figures 6A, 6B). The dose profile shift was mitigated at 5.0 mm (Figure 6C). Notably, when MLC compensation was activated for setup errors along the IEC-X direction, a shift in the dose profile was noted along the IEC-Z direction, despite no setup errors noted in the IEC-Z direction (Figure 6I).

**Figure 7:**
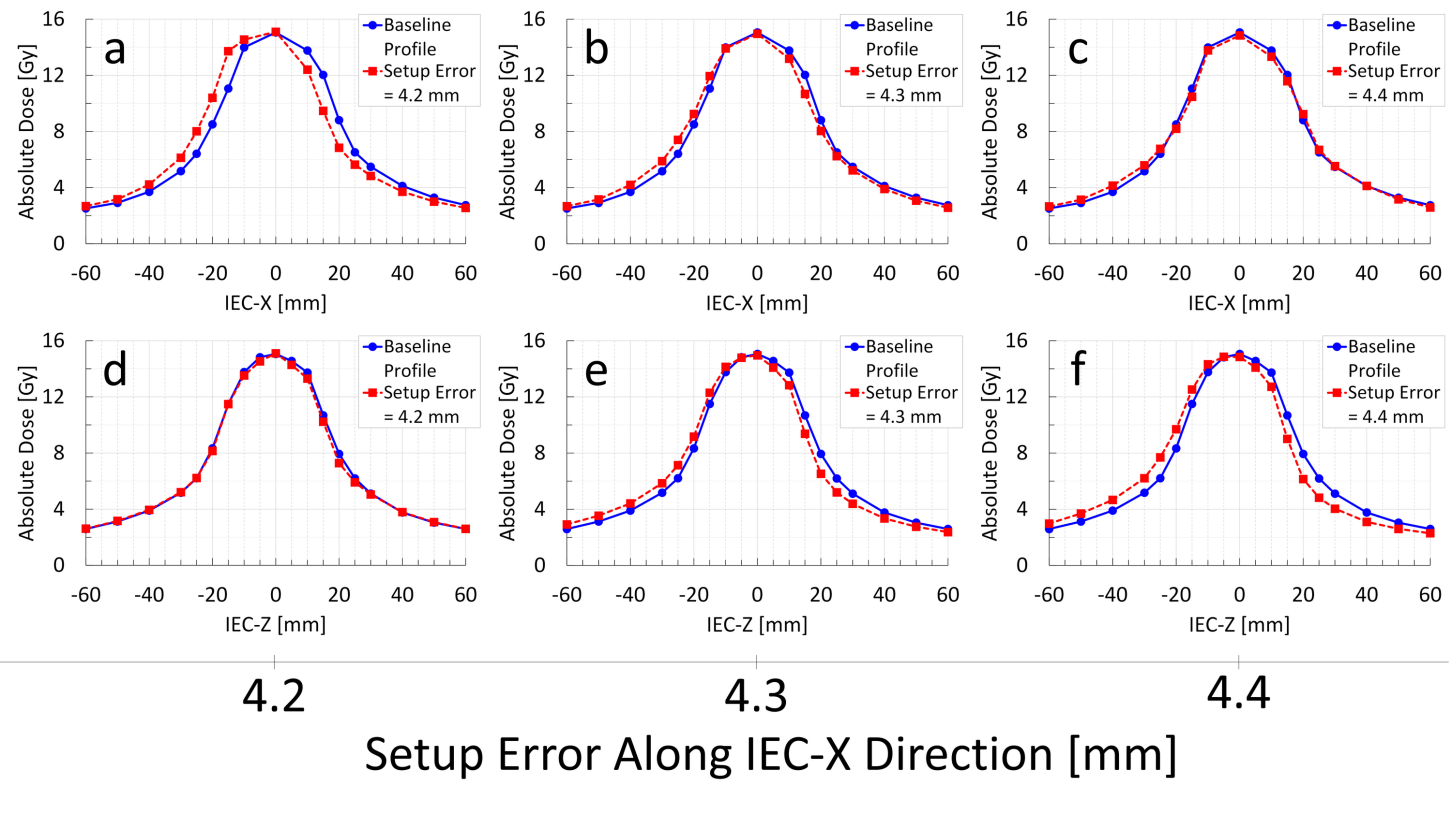
Change in Radixact Synchrony's response to target setup errors along the IEC-X direction: detailed investigation of MLC compensation effects via measured dose profiles through the center of the Delta4 Phantom+ (M0S0E0 vs. M1S1E1) The blue solid line indicates the baseline dose profile (M0S0E0), whereas the red dashed line represents the dose profile of Radixact Synchrony irradiation in response to a target setup error along the IEC-X direction (M1S1E1). The observed changes in the dose profile indicate that the IEC-X profile is corrected when the setup error reaches 4.3 mm (b). Despite no setup errors observed in the IEC-Z direction, a profile shift is noted in IEC-Z when the target setup error in IEC-X reaches 4.3 mm (e). These findings suggest that the binary MLC compensation threshold is approximately 4.3 mm.

To further evaluate the threshold at which MLC compensation occurs, additional measurements with finer setup error increments along the IEC-X direction were performed. Along the IEC-X direction, the dose profile remained shifted for setup errors of up to 4.2 mm; however, compensation was observed at 4.3 mm, which became more prominent at 4.4 mm (Figures 7A-C). In contrast, along the IEC-Z direction, where no setup error was introduced, the dose profile remained largely consistent for setup errors of up to 4.2 mm; however, slight deviation was observed at 4.3 mm, which became more evident at 4.4 mm (Figures 7D-F).

**Figure 8:**
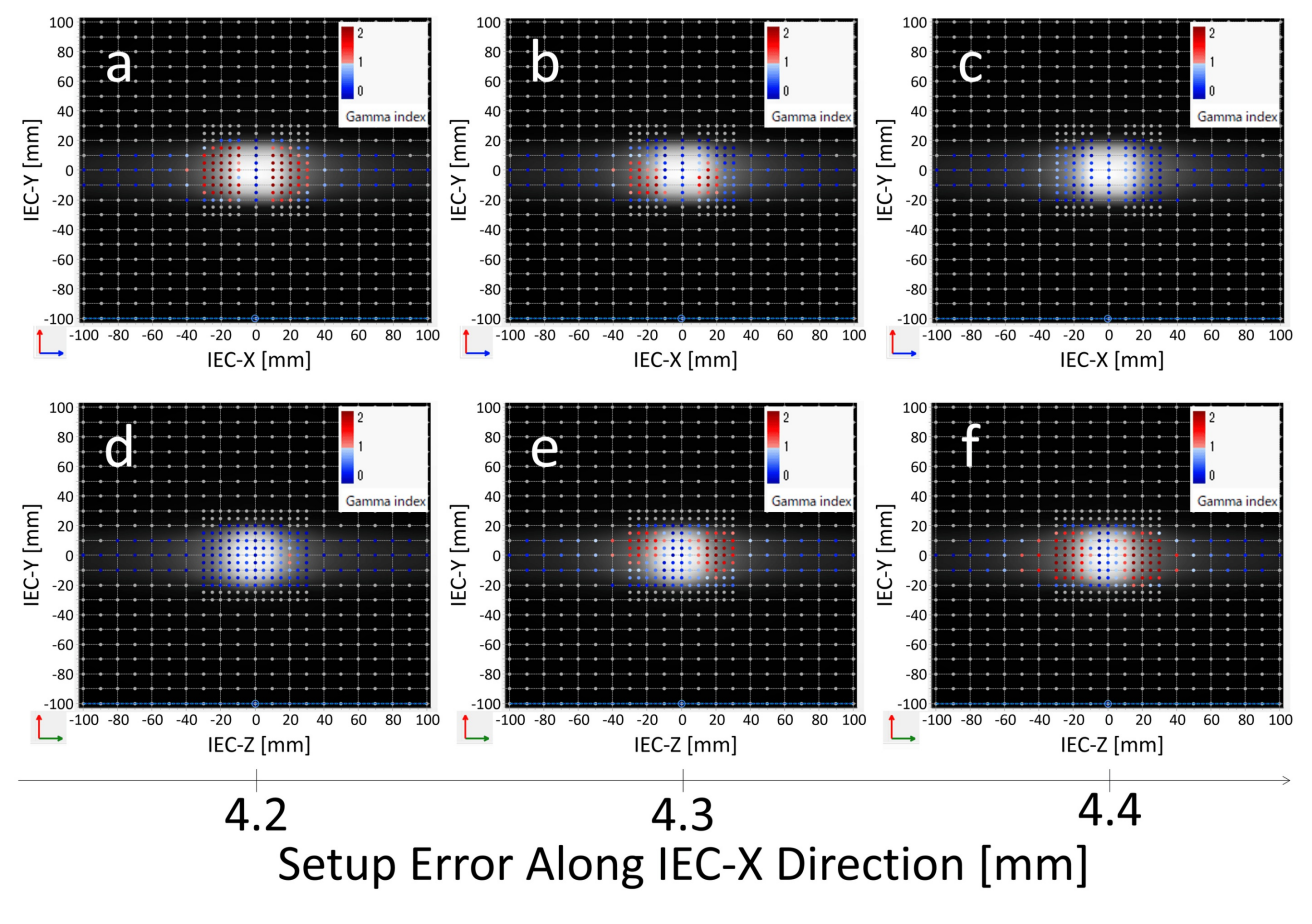
Change in Radixact Synchrony's response to target setup errors along the IEC-X direction: detailed investigation of MLC compensation effects via gamma index maps (M0S0E0 vs. M1S1E1) Gamma index maps were generated using Delta4 software to assess the dosimetric impact of target setup errors along the IEC-X axis. Panels (a–c) show the gamma distributions in the coronal plane for setup errors of 4.2 mm, 4.3 mm, and 4.4 mm, respectively, while panels (d–f) depict the corresponding distributions in the sagittal plane. Gamma analysis was performed using the 3%/1 mm criterion with a 10% dose threshold. Points with γ ≤ 1 are indicated by blue dots, whereas failing points (γ > 1) are shown in red. A reduction in failing points at 4.3 mm (b) suggests the onset of MLC compensation near this threshold. Although no setup errors were introduced along the IEC-Z axis, gamma failures are observed in the sagittal plane when the IEC-X setup error reaches 4.3 mm (e). These findings suggest that the binary MLC compensation threshold is approximately 4.3 mm. Furthermore, compensation activated in one direction appears to influence dose distribution in the orthogonal direction (c, f).

To visualize the spatial impact of MLC compensation in greater detail, gamma index maps corresponding to the setup errors in Figure 7 are presented in Figure 8. These maps further support the observation that MLC-based compensation is initiated at a threshold of approximately 4.3 mm. Specifically, the reduction in failing points on the gamma maps at 4.3 mm indicates the onset of compensation along the IEC-X axis (Figure 8B). Interestingly, despite the absence of setup errors along the IEC-Z axis, gamma failures in the sagittal plane become apparent at this threshold (Figure 8E), suggesting that MLC adjustments along one axis may influence dose distribution in the orthogonal direction (Figures 8C, 8F). These findings delineate not only the threshold at which binary MLC compensation is triggered but also its multidirectional dosimetric implications.

## Discussion

Radixact Synchrony system and motion correction mechanism

Our findings suggest that the Radixact Synchrony system exhibited a high level of robustness against setup errors along the Y direction. However, when a setup error developed in the transverse direction, the GPR decreased when the setup error was below the MLC compensation threshold but recovered upon MLC compensation activation (Figure 4). These findings indicate that although the Radixact Synchrony system offers precise target tracking and motion correction in the IEC-Y direction (corrected by the jaws), a compensation threshold exists in the transverse direction, with the MLC compensation mechanism engaging beyond a specific threshold. While Chen et al. [2] confirmed the accuracy of Synchrony for larger target displacements (≥5 mm), particularly using MLC compensation in the transverse directions, they did not assess system performance near the compensation threshold. Our study extends this understanding by focusing on target setup errors smaller than 5 mm, which were not covered in their evaluation. We observed that these sub-threshold displacements are not actively corrected by the MLC system, leading to a temporary degradation in GPR and dose distribution quality until the compensation threshold is exceeded (Figures 4, 8). Ferris et al. [3] reported that motion interplay and dose blurring were not observed in any of the cases studied when Synchrony tracking was enabled. While their findings emphasize the system's efficacy under active tracking, potential limitations under conditions where motion correction might not be triggered were not discussed. The present study seeks to address this limitation by providing evidence that, in the transverse directions, dose degradation may occur when setup errors remain below the compensation threshold, even with Synchrony enabled. These findings suggest that setup errors along the transverse directions can potentially influence irradiation accuracy, even in the presence of an active tracking system.

When the MLC compensation was applied in one direction, the dose distribution in the orthogonal direction was also influenced (Figures 6-8). This change is likely due to the rotational irradiation of the Radixact treatment, where the 6.25-mm-wide MLC compensation influenced both components in the axial plane (IEC-X and IEC-Z directions). Therefore, dose distribution may be influenced by setup errors along the transverse direction that reach the threshold owing to the effects of discrete compensation. Consequently, this spatial interplay underscores the complexity of the binary MLC compensation mechanism and highlights the importance of recognizing its multidirectional dosimetric effects when setup errors approach the system’s compensation threshold.

It should be noted that this study employed a phantom model, which allowed controlled analysis but does not replicate respiratory irregularities such as irregular motion or baseline drifts that may occur clinically. The Hexamotion system simulated target motion along the IEC-Y direction using a basic respiratory waveform designed to mimic respiratory-induced tumor motion. This simplified setup was intentionally chosen to estimate the threshold for MLC compensation under well-defined conditions. Accordingly, this setup represents a scenario where the dosimetric impact of MLC compensation becomes most evident when a transverse offset occurs. In particular, if a target offset develops along the transverse direction during irradiation, as may occur due to periodic respiratory motion or baseline shifts or drifts, the activation and dosimetric effect of MLC compensation may vary depending on the magnitude of the target motion and the duration for which the target remains offset. Consequently, while our results provide valuable insights into the tracking system's performance, their generalization to clinical practice, where more complex and multidimensional motion is present, requires further validation.

Another limitation of this study is that repeated measurements were not performed for each condition. Nevertheless, all experimental setups were conducted under tightly controlled conditions.

Binary MLC compensation threshold

The MLC compensation threshold was approximately 4.3 mm when the planned target position was at the IC. Analysis of dose profiles in the presence of an IEC-X setup error exhibited remarkable dose distribution shifts once the target setup error reached this threshold, indicating MLC compensation initiation (Figures 7, 8). Our findings offer additional insights into the compensation threshold and its impact on treatment accuracy.

MLC compensation is applied when the target motion exceeds 3.125 mm, and the discrete nature of the MLC leaf width (6.25 mm) contributes to overcompensation, thereby affecting the correction accuracy [6]. To address this issue, the current system is presumed to have adopted an approximately 4.3-mm threshold to reduce overcompensation. In cases where periodic respiratory motion develops, such as in lung or liver cancer, particularly when the target moves in the axial plane (IEC-X and IEC-Z directions), setting the MLC compensation threshold to 3.125 mm may result in frequent overcompensation whenever this threshold is exceeded. Therefore, the threshold adjustment is probably aimed at preventing a decline in irradiation accuracy.

This compensation threshold of approximately 4.3 mm was derived from measurements using a unidirectional (IEC-X) target offset and represents the system's recognized motion magnitude that triggers MLC compensation. No clear shifts in dose profiles indicative of MLC compensation were observed for setup errors smaller than this threshold, suggesting such offsets remained below the system's activation limit. While the beam's eye view (BEV) offset varies continuously with gantry angle during helical delivery, this study did not evaluate MLC compensation behavior at individual gantry angles.

The estimated MLC compensation threshold was derived on the basis of the planned target position at the IC. However, owing to beam divergence, the Cartesian distance corresponding to a shift of one leaf relies on the distance from the source. Consequently, the effective compensation threshold is theoretically subject to variation when the planned target position is far from the IC; however, further investigation is necessary to confirm the extent and clinical significance of this variation.

Clinical considerations: PTV margin in Radixact Synchrony

Our findings indicate that jaw tracking correction effectively maintained the GPR at >95% for IEC-Y setup errors while preserving the dose profile stability (Figures 4, 5). This finding suggests that dosimetric accuracy can be preserved even with relatively small PTV margins in the IEC-Y direction. In contrast, binary MLC compensation is activated in the transverse direction when the target setup error reaches 4.3 mm, thereby mitigating significant positional deviations in the dose distribution (Figures 7, 8). These results suggest that in the transverse direction, where MLC compensation is active, a 5-mm PTV margin is appropriate for ensuring adequate dose coverage for the gross tumor volume. Smaller margins (<5 mm) require more careful evaluation.

## Conclusions

This study evaluated the correction accuracy and dosimetric impact of the Radixact Synchrony system under conditions with target setup errors. The system maintained a high GPR without setup errors, confirming the reliability of its motion-tracking mechanism, and showed high robustness even with setup errors in the IEC-Y direction. However, setup errors along the IEC-X and IEC-Z axes revealed a compensation threshold of the binary MLC, resulting in dose distribution alterations. Our analysis indicates that this MLC compensation threshold is approximately 4.3 mm when the planned target position is at the IC. Although MLC compensation contributed to restoring GPR once the error exceeded the threshold, this mechanism should not be relied upon as a substitute for precise initial patient setup. The discrete nature and activation threshold of the MLC system impose inherent limitations on correction accuracy. These findings provide valuable insights into the position correction mechanism of the Radixact Synchrony system and its implications for treatment precision.
